# Clinical Outcome of Epilepsy Surgery of The Temporal Lobe in Paediatric Group: A Retrospective Cohort Study

**DOI:** 10.21315/mjms-12-2024-958

**Published:** 2024-12-31

**Authors:** Mohd Khairun Mohd Mispan, Azmi Alias, Ahmad Rithauddin Mohamed, Fauziah Kassim, Normawati Mat Said, Jafri Malin Abdullah

**Affiliations:** 1 Department of Neurosurgery, Tunku Abdul Rahman Neuroscience Institute, Hospital Kuala Lumpur, Kuala Lumpur, Malaysia; 2 Department of Paediatric Neurosurgery, Hospital Tunku Azizah (Women and Children Hospital), Kuala Lumpur, Malaysia; 3 Department of Paediatric (Paediatric Neurology), Hospital Tunku Azizah (Women and Children Hospital), Kuala Lumpur, Malaysia; 4 Department of Pathology, Hospital Kuala Lumpur, Kuala Lumpur, Malaysia; 5 Department of Paediatric (Paediatric Radiology), Hospital Tunku Azizah (Women and Children Hospital), Kuala Lumpur, Malaysia; 6 Department of Neurosciences, School of Medical Sciences, Universiti Sains Malaysia, Health Campus, Kelantan, Malaysia; 7 Brain Behaviour Cluster, Universiti Sains Malaysia Specialist Hospital and School of Medical Sciences, Universiti Sains Malaysia, Health Campus, Kelantan, Malaysia

**Keywords:** epilepsy surgery, Engel Outcome Classification, paediatric group, hippocampal sclerosis, tumour

## Abstract

**Background:**

This study was to identify seizure outcome following epilepsy surgery in paediatric group using Engel classification. We also identified histological and radiological findings of the study population.

**Methods:**

This study was a retrospective cohort study over a period of eight years from 1 January 2012 until 1 July 2019. Engel Outcome Classification was used to identify seizure outcome at immediate, three to six months and one year post-operation. Age, sex, age of seizure onset, age of operation, body weight, number of antiepileptic medications pre and post-operation, duration of stay in intensive care unit (ICU) and ward, magnetic resonance imaging (MRI) and histopathological findings and complications were noted. Analytically, Fisher’s exact test was used for categorical data. Wilcoxon Signed Rank test was used for continuous but not normally distributed data. In this study, Univariate logistic regression is used to understand whether Engel Classification I can be predicted from categorical variables and numerical variables. A set *P*-value of < 0.05 was considered of statistical significant.

**Results:**

29 paediatric patients met study criteria. The mean time between seizure onset and surgery was 5.6 years. Non-invasive evaluation was used in all 29 patients during preoperative assessment. With the use of the Engel Outcome Classification, Engel I outcome or seizure free was achieved in 28 patients (96.6%) at immediate follow-up, 24 patients (82.8%) at three to six months follow-up and 24 (82.8%) at one year follow-up. Hippocampal Sclerosis was present in 13 patients (44.8%). Tumours were present in 16 patients (55.2%). The complication proportion was 14.4%. Only two patients (6.9%) underwent reoperations. There are significant changes in post operative Anti Epileptic Medications (AEDs) reduction in this study (*P* = 0.006). Possible risk factor of seizure was suggestive for less recurrence of seizure, which may become a predictor to achieve Engel I classification in this study (*P* = 0.052).

**Conclusion:**

The surgery outcome of epilepsy surgery of the temporal lobe in paediatric group was comparable in this study. Careful patient selection by multidisciplinary evaluations is mandatory.

## Introduction

Epilepsy is a group of neurological disorders characterised by an increased tendency to develop epileptic seizures (1–3). Epileptic seizures are episodes that can vary from brief and nearly undetectable periods to long periods of vigorous shaking. The classification of epilepsy focuses on the underlying causes. The new classification of the Epilepsies is a multilevel classification, designed to cater to classifying epilepsy in different clinical environments (2, 4):

- Seizure types: focal onset, generalised onset, unknown onset
- Epilepsy types: focal, generalised, combined generalised and focal, unknown
- Epilepsy syndrome

The aetiology of the individual’s epilepsy includes structural, genetic, infectious, metabolic, autoimmune and unknown (2, 4).

Refractory epilepsy occurs when a person has failed to become seizure free with adequate trials of two seizure medications according to International League Against Epilepsy (ILAE) (2, 4). These seizure medications must have been chosen appropriately for the person’s seizure type, tolerated by the person and tried alone or together with other seizure medications.

Temporal lobe epilepsy (TLE) is a chronic disorder of the nervous system characterised by recurrent, unprovoked focal seizures that originate in the temporal lobe of the brain. TLE is the most common form of epilepsy with focal seizures (5, 6). A focal seizure in the temporal lobe may spread to other areas in the brain, and it may become a focal to bilateral seizure. The ILAE recognises two main types of TLE: mesial TLE arising in the hippocampus, the parahippocampal gyrus and the amygdala, which are located in the inner medial aspect of the temporal lobe and lateral TLE arising in the neocortex at the outer lateral surface of the temporal lobe (6–8).

The risk factors or causes of TLE include mesial temporal sclerosis, traumatic brain injury, febrile seizures, cerebral tumour, hypoxic brain injury, meningitis, stroke or non-identified (6–8). There is a link between febrile seizures and subsequent TLE, at least epidemiologically (7, 8).

TLE can be managed medically or surgically. Despite the usage of multiple antiepileptic drugs (AEDs), 20% to 25% of patients of all ages still have refractory epilepsy, and in the paediatric population, only 20% of patients have epilepsy of a temporal lobe origin (3, 8, 9). These patients have significant morbidity and increased lifetime mortality. Temporal lobe surgery in selected patients is one of the most effective treatments to obtain seizure control (8–11).

Reported seizure freedom after surgery in TLE in the paediatric population ranges from 60% and 86% (12–15), with a tendency to proceed with early surgical intervention in order to reduce the developmental morbidity of epilepsy and epileptic drugs (10, 16–18).

Lopez-Gonzalez et al. (19) reported that the surgical outcome with Engel classification at last follow-up resulted in 75% of patients being classified as class 1 (seizure free), and overall 84% of patients had a favourable outcome, class 1 and 2 (10, 18, 19). Long-term seizure control is achieved successfully with acceptable complication rates (10, 18, 19). Benifla et al. (20) reported 74% of patients had an Engel Class 1 or 2 surgical outcome (10, 18, 20). Benifla et al. (20) summarised that temporal lobe resections for epilepsy in children are effective and safe procedures with a favourable impact on seizure control (3, 8, 20).

The aim of surgical resection is to control or eliminate the seizure recurrence postoperatively. The seizure outcome is assessed by using the Engel classification system, which has become the standard in reporting postoperative outcomes of epilepsy surgery (21–23). Engel classification was devised by UCLA neurologist Jerome Engel Jr. in 1987 and made public in 1992. An advantage of Engel classification is its subjectivity as it leaves much of the postoperative class assignment process to the patients. This system involves patients or family members in determining the postoperative evaluation (3, 8, 24). Engel classification allows seizure outcome assessment up to two years postoperatively. One disadvantage of Engel classification is that there is no quantitative definition of what determines a rare occurrence or a method to measure worthwhilesness (3, 8, 24). Another seizure outcome classification is the ILAE classification, which was developed in 2001. It was proposed due to the disadvantages of Engel classification. ILAE classification reports outcome based upon frequency of postoperative seizure days and does not include subjective classification of seizure control or quality of life, theoretically making it more objectively measured (3, 8, 24). Of note, there are no specific outcome scales that are widely used, but both systems should facilitate collaboration and comparison between centres (3, 8, 25). Both classifications have very good inter-rater reliability in the paediatric population undergoing respective surgery for refractory epilepsy. In this study, Engel classification is used as most outcomes using this classification have been classified retrospectively. The ILAE system was strongly advocated for prospective studies (3, 8, 24).

Even with available data, there is still reluctance to refer paediatric patients for epilepsy surgery evaluations (3, 8, 26, 27). In many countries, the resources to perform such evaluations and procedures are not widespread (3, 8, 28, 29). In Malaysia, cultural perception of brain surgery is also a key barrier, for which most Malaysians perceived brain surgery as a major surgery with high morbidity and mortality (29, 30).

In this study, the surgical experience in a single institution, Hospital Kuala Lumpur, by a single neurosurgeon, is analysed to further understand the role of epilepsy surgery of the temporal lobe in this subset of paediatric patients. Of note, Hospital Kuala Lumpur is the only Ministry of Health Malaysia hospital in this country that offers such procedures to paediatric groups. There is lack of data about the outcome of this group since the surgical procedure was initiated in Hospital Kuala Lumpur in 2012. Hopefully, by providing local data, the public will be more convinced of this option of treatment for TLE.

## Methods

This retrospective cohort study was conducted from 1 January 2012 to 1 July 2019. Ethical approval has been obtained from the Medical Research and Ethics Committee of the Ministry of Health Malaysia (approval number NMRR-19-1880-48547) and the Human Research Ethics Committee of Universiti Sains Malaysia (approval number USM/ JEPeM/21080578).

All paediatric patients who underwent temporal lobectomy or lesionectomy for epilepsy surgery at Hospital Kuala Lumpur were reviewed. Operations were performed by a single senior consultant neurosurgeon. Resective surgical techniques included anteromesial temporal lobectomy (ATL) or lesionectomy.

The patients were evaluated by a consultant paediatric neurologist, and preoperative evaluations were completed in a standard fashion. The cases were discussed at epilepsy multidisciplinary team (MDT) meetings comprising a paediatric neurologist, a neurosurgeon and a neuroradiologist. Preoperative evaluations included detailed clinical history, seizure semiology, frequency of seizures and response to AEDs. Data discussed included electroencephalography (EEG) monitoring and magnetic resonance imaging (MRI) with epilepsy protocol. Recommendations of the MDT committee were then discussed with the patients and their families, and informed consent was obtained.

### Resective Surgical Procedures

The surgical approach was selected according to the MRI findings, EEG data and preoperative diagnosis. The surgical techniques performed were as follows:

#### ATL

This procedure involved the removal of a portion of the lateral temporal cortex and the mesial temporal lobe structures (amygdala, hippocampus and parahippocampus) as a two-part process. It was performed in patients with mesial temporal lobe pathology and those with pathology in both the lateral and mesial temporal lobe compartments. The extent of the lateral temporal resection was determined by the senior consultant neurosurgeon, averaging 3.5 cm on the dominant side and 4.5 cm on the non-dominant side. Variations were based on cortical vascular anatomy and tailored in lesional cases to include the posterior extent of the lesion (8, 10, 31).

#### Lesionectomy

This procedure involved the resection of the lesion (neoplasm) identified on preoperative imaging. The extent of resection was typically determined by intraoperative findings, with or without the use of intraoperative ultrasound (8, 10, 31). For this group of patients, the mesial temporal lobe structures were not included.

#### Lesionectomy + ATL

This procedure extended beyond the ATL described above also to include a lesion, usually a tumour identified on preoperative MRI (8, 10, 31).

#### Selective Amygdalohippocampectomy

This procedure involved the resection of mesial temporal lobe structures via a transcortical approach through the middle temporal gyrus. This technique is indicated for cases with a clearly defined unilateral mesial temporal epileptogenic zone, thus limiting the extent of lateral neocortical resection (8, 10, 31).

All patients received standardised postoperative care during the immediate period, including one day of intensive care unit (ICU) observation and the continuation of AEDs. Postoperative seizure outcomes were assessed using the Engel classification scale at three time points: immediately post-operation, at the first follow-up (three to six months) and annually thereafter. AED dependency was also retrospectively evaluated.

Preoperative imaging included a brain MRI. Postoperatively, either a CT scan or an MRI was performed. Dates of all imaging studies (pre and postoperative) and surgery were identified retrospectively. Neurohistopathological diagnoses (hippocampal sclerosis [HS] and tumours) were obtained for each patient from the Department of Pathology records. Surgical complications, if any, such as wound infection, visual field defect, hemiparesis, status epilepticus, diplopia and cerebrospinal fluid leakage were identified and recorded.

Patients’ demographic data were also obtained. These include gender, ethnicity, age at seizure onset, age at surgery, time between age at seizure onset and age at surgery, seizure duration, weight, operation hours, percentage of blood loss, postoperative length of stay and number of medications administered pre and post-operation.

Patients: i) ≤ 18 years of age, ii) with refractory epilepsy despite being on at least two AEDs and iii) with MRI findings indicative of epilepsy were included. However, those with extratemporal lobe epilepsy, such as the frontal, parietal or occipital lobe epilepsy, were excluded.

The primary endpoint of this study was to determine patient outcomes using the Engel classification scale. The outcomes were assessed at three time points: immediately post-operation, at the first follow-up (three to six months) and annually thereafter. The patients’ outcomes were recorded by the consultant paediatric neurologist and subsequently collected by the primary investigator. The secondary endpoint was to identify the histopathological and radiological findings of the study population. The tertiary endpoint was to identify potential prognostic factors for satisfactory seizure outcome (Engel Class I).

Data were analysed using SPSS version 20. Descriptively, all categorical data were expressed as frequencies and percentages, while continuous data were expressed as mean ± standard deviation (SD) or as median (interquartile range), whichever was appropriate. Analytically, Fisher’s exact test was used for categorical data. If the data were continuous and normally distributed, a *t*-test was used. If the data were continuous but not normally distributed, the Wilcoxon Signed Rank test was used. Univariate logistic regression was used to determine whether Engel Classification I status can be predicted by various categorical and numerical variables. A two-tailed *P*-value of <0.05 was considered to be statistically significant for all analyses.

## Results

### Demographic and Preoperative Evaluation

Between 1 January 2012 and 1 July 2019, 33 paediatric patients underwent epilepsy surgery. Four of these patients had extratemporal epilepsy and were therefore excluded from the study. Table 1 summarises the demographics of the population studied. The remaining 29 patients included 16 males (55.2%) and 13 females (44.8%). The majority of patients were Malay (*n* = 28, 96.6%), followed by Chinese (*n* = 1, 3.4%). All 29 patients (100%) were right handed.

Identified possible risk factors for epilepsy were as follows: none, 17 (58.6%); febrile seizure, five (17.2%); dengue haemorrhagic fever, one (3.4%); duplex kidney, one (3.4%); family history of epilepsy, one (3.4%), meningoencephalitis, one (3.4%); history of recurrent neonatal apnoea, one (3.4%); strong family history of childhood seizure, one (3.4%); and non-accidental injury post-trauma, one (3.4%).

The mean ages at seizure onset and at surgery were 6.6 years (SD ± 4.2) and 12.1 years (SD ± 4.08), respectively. The mean interval between age at seizure onset and age at surgery was 5.6 years (SD ± 3.68). The mean number of medications taken preoperatively was 2.4 (SD ± 0.78).

Patients had between one and five AED trials before surgical treatment (mean 2.4). At least two AED trials failed in all patients with chronic epilepsy, except for two (6.9%) patients who underwent surgery after one trial because of preoperative imaging features indicating a tumour or neoplasm.

All 29 patients (100%) had non-invasive evaluations and MRI as an investigation preoperatively. Twenty patients (69.0%) had electroencephalogram (EEG) investigation done preoperatively. Six patients (20.7%) had EEG and positron emission tomography (PET). One patient (3.4%) had EEG, PET scan and video EEG. Two patients (6.9%) had MRI as the only investigation done preoperatively. Preoperative MRI findings are shown in Table 2.

### Surgical Procedures

The surgical procedures are presented in Table 3.

The surgical side was right in 10 patients (34.5%) and left in 19 patients (65.5%). ATL surgery was performed in 25 patients (86.2%), while lesionectomy was performed in four patients (13.8%). None of the patients required an awake craniotomy.

The average estimated blood loss was 12.0% of body weight (SD ± 9.25), and the average surgical time was 4.9 h (SD ± 0.68). The average weight of patients was 47.1 kg (SD ± 19.92). All patients (100%) received a postoperative CT scan on day one to rule out immediate complications such as bleeding or infarction.

Postoperative MRI was performed in 14 patients (41.38%) three to six months postoperatively. Analysis of these studies demonstrated complete resection in 12 patients (85.71%). Two patients (14.28%) had incomplete resection and subsequently underwent reoperation.

### Neuropathology Findings

The pathology findings (Table 4) were divided into two main groups: the HS group (*n* = 13, 44.8%) and the tumour group (*n* = 16, 55.2%).

**Figure 1:**
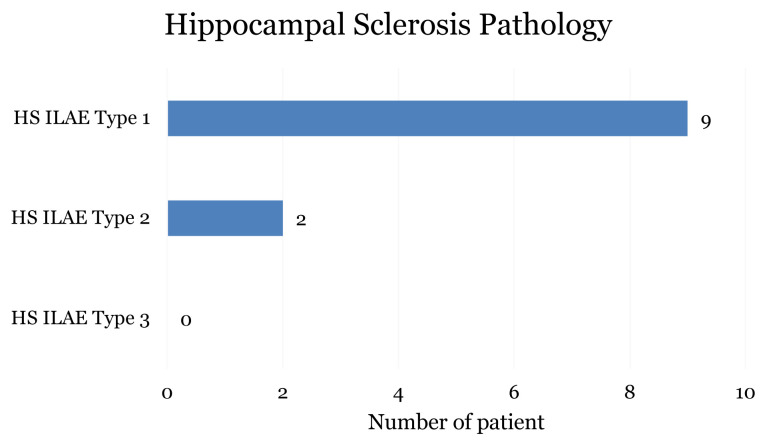
HS pathology

For 13 patients in the HS group, following the International Consensus Classification of Hippocampal Sclerosis (32, 33) in TLE, nine patients (69.23%) were classified as Hippocampal Sclerosis International League Against Epilepsy (HS ILAE) Type 1, and two patients (15.38%) were classified as HS ILAE Type 2. No patients were classified in the HS ILAE Type 3 group (Figure 1). The remaining two patients (15.38%) were diagnosed with “mild HS” histologically because their findings did not fulfil the criteria of any of the three types of HS ILAE.

HS ILAE Type 1 refers to severe neural cell loss and gliosis predominantly in CA1 and CA4 regions. HS ILAE Type 2 shows predominant neural cell loss and gliosis in the CA1 region. Lastly, HS ILAE Type 3 demonstrates predominant neural cell loss and gliosis in the CA4 region.

### Outcome

**Figure 2:**
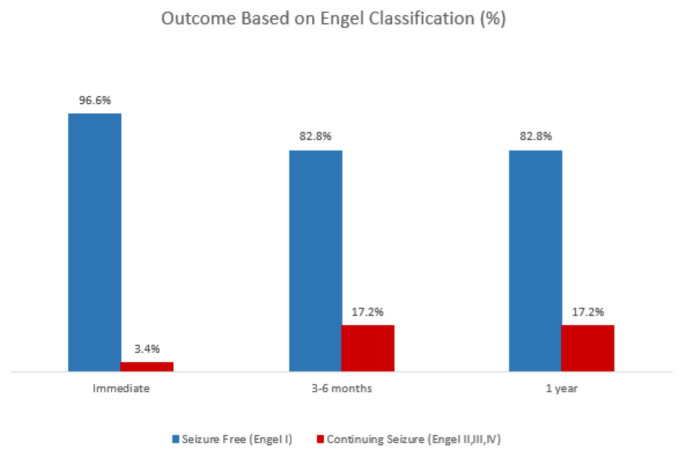
Surgical outcome based on Engel classification

Using the Engel Outcome Classification (Tables 5 and 6, Figure 2), immediate follow-up revealed that 28 patients (96.6%) were Class I, and one (3.4%) was Class IV. Precisely, 28 patients were seizure free, and one had continuing seizures.

At the three to six month follow-up, 24 (82.8%) were Class I, three (10.3%) Class II and two (6.9 %) Class IV. This suggested that 24 patients were seizure free, and 5 (17.2%) had continuing seizures during the follow-up period.

At one year follow-up, 24 (89.7%) were Class I, three (10.3%) Class II and two (6.9%) Class IV. That is, 24 (82.8%) were seizure free, and five (17.2%) had continuing seizures during the follow-up period.

Postoperatively, all 29 patients (100%) had a one day stay in the ICU. The mean length of stay in the normal ward postoperatively was 4.4 days (SD ± 2.98). The mean follow-up duration for all patients after surgery was 770.2 days (SD ± 637.07).

### Correlation between Prognostic Factors and Seizure Outcome

Fisher’s exact test was used to determine the association between categorical prognostic factors and seizure outcome. Gender, tumour aetiology, side of surgery and number of AED trials were not found to be statistically significant. However, a potential association was observed for one factor regarding less seizure recurrence and achieving an Engel I classification, though it did not reach the pre-defined level of statistical significance (*P* = 0.052) (Table 7).

### Prognostic Factors for Seizure Outcome

Univariate logistic regression analysis revealed that age at surgery, age at seizure onset, time between age at seizure onset and age at surgery, weight, blood loss, operation hour, gender, ethnicity, tumour aetiology, ATL, side of surgery, residual and number of AED trials were not significantly associated with the outcome. No potential risk factors were found to be significantly associated with Engel I outcome classification. Multivariate analysis revealed no significant findings (Tables 8 and 9).

### Study on HS and Tumour Group

Table 10 presents the characteristics of the study population categorised by neuropathological findings, specifically HS and tumour.

**Figure 3:**
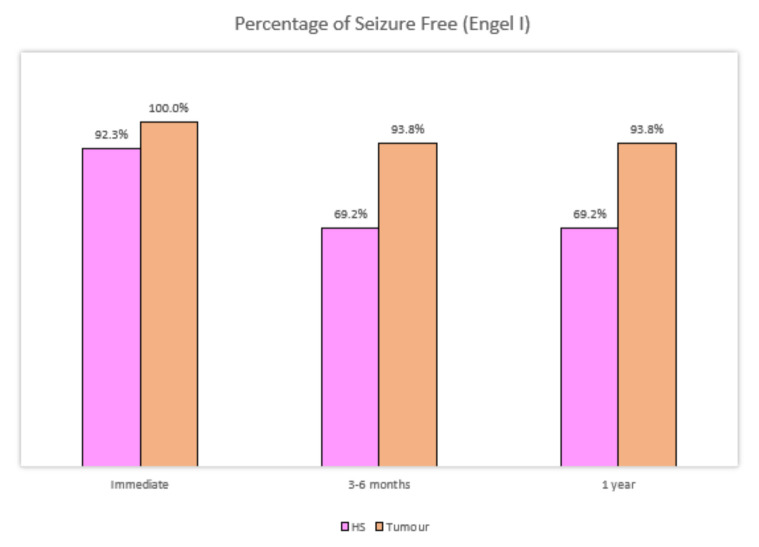
Percentage of seizure free (Engel I) between the HS and tumour groups

Table 11 and Figure 3 depicts the outcome cross-tabulation. The Engel I outcome for the HS group at immediate, three to six months, and one year follow-up was achieved by 12 (92.3%), nine (69.2%) and nine (69.2%) patients, respectively. For the tumour group, the corresponding Engel I outcomes were 16 (100%), 15 (93.8%) and 15 (93.8%) patients, respectively.

### Differences between HS and Tumour Group

Independent *t*-test and Mann–Whitney U test were used to determine whether there was any significant difference in age at surgery, age at seizure onset, weight, blood loss, number of medications administered pre-operation, number of medications administered post-operation and length of stay after operation between the HS and tumour groups. Based on Table 12, only the number of medications postoperatively was statistically significant. Therefore, we can conclude that there is a significant difference in the number of epileptic medications post-operation between the HS and tumour group in this study.

### Complication

Complications were present in four (14.4%) patients out of 29 patients (Table 13).

### Other Findings

#### Association Between the Number of Antiepileptics Before and After Operation

From Spearman’s correlation analysis, it is shown that there is an association between the number of epileptic medications before and after operation (Table 14). The Spearman’s correlation coefficient is 0.427 with a *P*-value of 0.021. This indicates that there is a weak positive linear relationship between the number of epileptic medications before and after operation.

#### Reduction in the Number of AEDs After Operation

In this study, the number of AEDs postoperatively was reduced. There is a significant difference in the number of epilepsy medications before and after one year of operation. The Wilcoxon Signed Rank test showed that the number of epileptic medications after operation is reduced as compared to the number of epileptic medications before operation (*P* = 0.006) (Table 15).

## Discussion

### Surgical Procedures and Outcome

Wiebe et al. (12) study found that patients with epilepsy arising from the temporal lobe are among the best candidates for surgical intervention (8, 10, 12). Hence, this option of treatment should be considered when seizures persist despite optimal medical therapy. In the adult group, this has been defined as the failure to respond to at least two AEDs, whether as monotherapies or in combination, with adequate therapeutic level (4, 32). But, this strategy may not apply to the paediatric group because of the risk of cognitive impairment if seizures persist (34, 35). Many studies have reported the success rate of temporal lobe surgery in the paediatric group (15, 27, 36, 37). Unfortunately, its utilisation has not been widespread in developing countries (27–29), including Malaysia. In our study, the mean age onset of seizure was 6.6 years (± SD 4.2), and the mean age at surgery was 12.1 years (± SD 4.08). This means the mean time between age onset of seizure and age at surgery was 5.6 years (± 3.68), and patients were having between one and five antiepileptic drug trials before surgical intervention (mean 2.4 ± SD 0.78).

Selection of surgical candidates required thorough evaluation of the clinical, electrophysiological, neuroimaging by multidisciplinary team including paediatric neurologist, radiologist and neurosurgeon. In this study, all 29 patients (100%) had MRI as an investigation. Twenty (69%) had EEG, seven (24.1%) had EEG and PET, as investigation preoperatively. None of the patients had invasive evaluation.

The classic neuroimaging findings of HS (hippocampal volume loss and increased signal on fluid attenuation inversion recovery) were demonstrated in 11 patients (37.9%). Two patients (6.9%) had temporal volume loss only from MRI. Two patients (6.9%) showed neocortical lesions, four patients (13.79%) showed amygdala or parahippocampal lesions, and 10 patients (34.48%) showed temporal lobe lesions from MRI. No normal MRI were seen in this study.

Our results indicate that at immediate, three to six months and one year, seizure freedom or Engel I outcome classification was reached in 96.6%, 82.8% and 82.8%, respectively. It can be seen that as time passes, the seizure freedom rate declines. This decline may be due to secondary epileptogenesis or incomplete resection of the epileptogenic zone. Overall, a favourable outcome was achieved in 82.8% (Engel Class 1) from the last follow-up (one year). This study shows that the outcome is better compared to the studies by Lopez-Gonzalez et al. (19) and Benifla et al. (20).

In this study, our most frequent finding was tumour 55.2% (16 patients), meanwhile HS was 44.8% (13 patients). For HS group, our results indicate that immediate, three to six months and one year Engel I outcome was 92.3%, 69.2% and 69.2% respectively. In comparison, the tumour group showed a better Engel I outcome at immediate, three to six months and one year, which was 100%, 93.8% and 93.8% respectively. In general, the tumour group shows better seizure freedom after operation in this study.

In our study, with Fisher’s exact test analysis, possible risk factor was suggestive for less recurrence of seizure, which may become a predictor to achieve Engel I classification (*P* = 0.052). However, from Univariate Logistic Regression Analysis, a possible risk factor was found as non-significant to associate with Engel I outcome classification.

In our study, there is a significant reduction of AEDs after one year follow-up (*P* = 0.006). Mean of number of AEDs preoperatively was 2.41 (± SD 0.78) meanwhile mean number of AEDs postoperatively was 1.93 (± SD 0.70). Discontinuing AED treatment after surgery is controversial, but it is usually recommended to wait for at least one year of seizure freedom and absence of epileptic activity on EEG (37, 38).

Our overall complication proportion was 14.4% (*n* = 4), including hemiparesis, mydriasis, ptosis and hemianopsia. Three (75%) out of four patients came from tumour group, one patient came from HS among those who had complications. Tumour group that have complications are due to tumour component that has calcified mesial component adjacent to and involving anterior choroidal artery. Another contributory factor for complications was early experience during the early years of performing these procedures. There was no mortality or long-term disabling morbidity. In previous series, the complication rates ranged from 2% to 8% (15, 39–42).

In this study, out of 29 patients, two patients (6.9%) had reoperation for completeness of surgical resection (Table 16).

### Pathology

Our most frequent finding was tumour, found in 55.2% (*n* = 16) of cases. All the tumours are low grade tumours which include ganglioglioma (*n* = 6), pleomorphic xanthoastrocytoma (PXA, *n* = 2), dysembryoplastic neuroepithelial tumour (DNET, *n* = 1), low grade astrocytoma (*n* = 3), oligoastrocytoma (*n* = 1), pilomyxoid astrocytoma (PMA, *n* = 1) and diffuse glioneural tumour (*n* = 2). In our study, the tumour group showed better seizure freedom at immediate, three to six months, and one year follow-up (100%, 93.8%, 93.8% seizure freedom at each follow-up, respectively).

Overall, HS was present in 44.8% (*n* = 13) of patients. Nine patients (69.2%) were classified as HS ILAE Type 1 (severe neural cell loss predominantly in CA1 and CA4 regions). Two patients (15.3%) were classified as HS ILAE Type 2 (predominant cell loss in CA1 region). Two patients (15.38%) were grouped as “mild HS” as histological findings did not fulfil the criteria of any three types of HS ILAE description.

## Conclusion

Engel Outcome Classification is a tool to assess the outcome of seizure freedom outcome in epilepsy surgery including the paediatric group. With the advancement of MRI and other modern technological advancements, namely EEG and PET scan, with careful selection of patients, the operation outcome has significantly improved the patients’ lives.

In this study, the outcome of epilepsy surgery of temporal lobe in the paediatric group was very good within a one year follow-up. The tumour group showed 93.8% of seizure freedom until last follow-up (at one year follow-up), meanwhile HS group showed 69.2% of seizure freedom until one year follow-up. There was no mortality throughout this study.

This study showed that the number of epileptic medications after operation is reduced as compared to the number of epileptic medications before operation (*P* = 0.006).

This was made possible due to a well-coordinated teamwork between the neurosurgeon, paediatric neurologist, neuroradiologist and neuropathologist.

### Limitations and Future Recommendations

As with all other studies, this study also has some limitations. The data obtained were descriptive and from a single institutional study. Apart from the small number of patients, statistical analysis could not be performed between certain groups since this is a descriptive analysis study.

For future study, it is recommended that more number of patients should be included so that more statistical study can be performed. Longer period of follow-up, like two years or more should be studied to review the outcome of the study. A prospective and a bigger sample size are proposed in order to address the limitation encountered in this study.

## Figures and Tables

**Table 1:** Characteristics of study population (*n* = 29)

Variable	*n*	Percentage (%)
Gender		
Male	16	55.2
Female	13	44.8

Ethnic		
Malay	28	96.6
Chinese	1	3.4

Handedness		
Right	29	1000.0
Left	0	0.0

Possible risk factors		
None	17	58.6
Febrile seizure	5	17.2
Dengue haemorrhagic fever	1	3.4
Duplex kidney	1	3.4
Family history of epilepsy	1	3.4
Meningoencephalitis	1	3.4
Recurrent neonatal apnoea	1	3.4
Strong family history of childhood seizure	1	3.4
Non-accidental injury post-trauma	1	3.4

Possible risk factors class		
No	17	58.6
Yes	12	41.4

Invasive evaluation		
Yes	0	0.0
No	29	100.0

MRI abnormalities		
Present	29	100.0
Absent	0	0.0

Other preoperative investigations		
EEG	21	72.4
EEG, PET	7	24.1
MRI only	1	3.5

HPE		
HS	13	44.8
Tumour	16	55.2

Complication		
None	25	86.2
Left hemiparesis	1	3.4
Right hemiparesis	1	3.4
Right hemiparesis, left mydriasis and ptosis	1	3.4
Right hemiparesis, right nasal hemianopsia	1	3.4

Number of epileptic medications		
1	2	6.9
2	15	51.7
3	11	37.9
4	0	0.0
5	1	3.4

Number of epileptic medications post-operation		
1	8	27.6
2	15	51.7
3	6	20.7

ICU stay postoperative (days)		
1	29	100

Length of stay in ward postoperative (days)		
3	18	62.1
4	7	24.1
10	2	6.9
12	1	3.4
14	1	3.4

MRI = magnetic resonance imaging; EEG = electroencephalogram; PET = positron emission tomography; HPE = histopathological examination; ICU = intensive care unit; HS = hippocampal sclerosis

**Table 2:** Preoperative MRI findings

Preoperative MRI findings	*n* (%)
Hippocampal volume loss with increased signal on T2/FLAIR	11 (37.93)
Neocortical lesion	2 (6.9)
Amygdala/parahippocampal lesion	4 (13.79)
Temporal lesion	10 (34.48)
Temporal volume loss	2 (6.9)

Total	29 (100)

MRI = magnetic resonance imaging

**Table 3:** Surgical procedures

Variables	*n*	Percentage (%)
ATL		
Left ATL	16	55.2
Right ATL	9	31.0
Left temporal lesionectomy	3	10.3
Right temporal lesionectomy	1	3.4

ATL		
ATL	25	86.2
Non-ATL	4	13.8

Side of surgery		
Right	10	34.5
Left	19	65.5

Total	29	100.0

ATL = anterior temporal lobectomy

**Table 4:** Neuropathology findings

Neuropathology findings	*n* (%)
HS	13 (44.8)
Tumour (Ganglioglioma, PXA, DNET, low grade astrocytoma, oligoastrocytoma, PMA, diffuse glioneural tumour)	16 (55.2)

PXA = pleomorphic xantoastrocytoma; PMA = pilomyxoid astrocytoma; DNET = dysembryoplastic neuroepithelial tumour; HS = hippocampal sclerosis

**Table 5:** Engel classification for outcome

Engel classification group	Frequency	Percentage (%)
Immediate		
Seizure free (Engel I)	28	96.6
Continuing seizure (Engel II, III, IV)	1	3.4

Three to six months		
Seizure free (Engel I)	24	82.8
Continuing seizure (Engel II, III, IV)	5	17.2

One year		
Seizure free (Engel I)	24	82.8
Continuing seizure (Engel II, III, IV)	5	17.2

**Table 6:** Engel I outcome

Engel I	*N*	Percentage (%) of seizure free group
Immediate	28	96.6
Three to six months	24	82.8
One year	24	82.8

**Table 7:** Factors associated with seizure outcome

Independent variable	Seizure free group (Engel I) one year	Continuing seizure group (Engel II, III, IV) one year	*P*-value
Gender			
Male	14	2	0.396
Female	10	3	

Possible risk factor			
No	12	5	0.052
Yes	12	0	

Side of surgery			
Right	9	1	0.424
Left	15	4	

Number of AED trials			
> 2	10	2	0.671
≤ 2	14 3		

HPE			
HS	9	4	0.107
Tumour	15	1	

* Significant if *P* ≤ 0.05;

Categorical data are presented as count; AED = antiepileptic drugs; HPE = histopathological examination; HS = hippocampal sclerosis

**Table 8:** Univariate seizure outcome predictors (univariate analysis table)

Independent variable	Seizure free group (Engel I) one year	Continuing seizure group (Engel II, III, IV) one year	B (SE)	Odds ratio	*P*-value
Age at surgery	12.23 (4.26)	11.580 (2.46)	0.039 (0.148)	1.039	0.794

Age on seizure	6.89 (4.26)	4.500 (3.5)	0.155 (0.169)	1.168	0.356

Time between on set and surgery	5.49 (3.67)	7.130 (4.39)	−0.113 (0.155)	0.893	0.466

Weight	46.56 (20.49)	52.000 (16.37)	−0.014 (0.032)	0.986	0.651

Blood loss	12.25 (9.72)	10.000 (3.0)	0.053 (0.137)	1.054	0.700

Operation hour	4.95 (0.70)	4.934 (0.52)	0.040 (0.911)	1.041	0.965

Gender					
Male	14	2	−0.742 (1.00)	0.476	0.459
Female	10	3			

Possible risk factor					
No	12	5 −20.327 (11,602.71)	0.000	0.999	
Yes	12	0			

Side of surgery					
Left	9	1	0.875 (1.195)	2.400	0.464
Right	15	4			

Number of AED trials					
> 2	10	2	−0.069 (1.002)	0.933	0.945
≤ 2	14	3			

HPE					
HS	9	4	−1.897 (1.198)	0.150	0.112
Tumour	15	1			

* Significant if *P* ≤ 0.05;

Numerical data are presented as mean (SD), while categorical data as count; AED = antiepileptic drugs; HPE = histopathological examination; HS = hippocampal sclerosis

**Table 9:** Multivariate analysis table

Independent variable	Multivariate analysis		

	B (SE)	Odds ratio	*P*-value
Age at surgery	−107.050 (93,469.89)	0.000	0.999

Age on seizure	120.798 (91,896.52)	2.897 × 10^52^	0.999

Time between on set and surgery	111.180 (91479.01)	1.924 × 10^48^	0.999

Weight	−2.120 (1,760.05)	0.111	0.999

Blood loss	−5.780 (5,478.46)	0.003	0.999

Operation hour	−3.560 (71,168.83)	0.028	1.000

Gender			0.998
Female		1.000	
Male	37.640 (16,487.38)	2.217 × 10^16^	

Possible risk factor			0.998
No		1.000	
Yes	−80.620 (28,925.94)	0.000	

Side of surgery			0.998
Right		1.000	
Left	58.200 (41,740.76)	1.891 × 10^25^	

Number of AED trials			0.998
≤ 2		1.000	
> 2	2.730 (71,673.98)	15.304	

HPE			0.998
HS		1.000	
Tumour	−43.310 (14,204.87)	0.000	

* Significant if *P* ≤ 0.05;

From multivariate analysis, there are no significant findings from this study

**Table 10:** Characteristics of the study population by neuropathological findings (HS and tumour)

HPE Group	HS		Tumour	

Variable	Frequency	Percentage (%)	Frequency	Percentage (%)
Gender				
Male	6	46.2	10	62.5
Female	7	53.8	6	37.5

Ethnic				
Malay	13	100.0	15	93.8
Chinese	0	0.0	1	6.3

Handedness				
Right	13	100.00	16	100.0
Left	0	0.0	0	0.0

Possible risk factors				
None	5	38.5	12	75.0
Febrile seizure	5	38.5	0	0.0
Dengue haemorrhagic fever	1	7.7	0	0.0
Duplex kidney	0	0	1	6.3
Family history of epilepsy	0	0	1	6.3
Meningoencephalitis	1	7.7	0	0.0
Recurrent neonatal apnoea	1	7.7	0	0.0
Strong family history of childhood seizure	0	0	1	6.3
Non-accidental injury post-trauma	0	0	1	6.3

MRI abnormalities				
Present	13	100.0	16	100.0
Absent	0	0	0	0.0

Other pre				
EEG	8	61.5	12	75.0
EEG, PET	4	30.8	3	18.8
MRI only	1	7.7	1	6.3

Operation				
Left ATL	7	53.8	9	56.3
Right ATL	6	46.2	3	18.8
Left temporal lesionectomy	0	0.0	3	18.8
Right temporal lesionectomy	0	0.0	1	6.3

ATL				
ATL	13	100.0	12	75.0
Non-ATL	0	0.0	4	25.0

Side of surgery				
Right	6	46.2	4	25.0
Left	7	53.8	12	75.0

Complication				
None	12	92.3	13	81.3
Left hemiparesis	0	0.0	1	6.3
Right hemiparesis	1	7.7	0	0.0
Right hemiparesis, left mydriasis and ptosis	0	0.0	1	6.3
Right hemiparesis, right nasal hemianopsia	0	0.0	1	6.3

Number of epileptic medications				
1	0	0.0	2	12.5
2	6	46.2	9	56.3
3	6	46.2	5	31.3
4	0	0.0	0	0.0
5	1	7.6	0	0.0

Number of epileptic medications post-operation				
1	1	7.7	7	43.8
2	8	61.5	7	43.8
3	4	30.8	2	12.5

Engel classification: Immediate				
Seizure free (Engel I)	12	92.3	16	100.00
Continuing seizure (Engel II, III, IV)	1	7.7	0	0.0

Engel classification: Three to six months				
Seizure free (Engel I)	9	69.2	15	93.8
Continuing seizure (Engel II, III, IV)	4	30.8	1	6.3

Engel classification: One year				
Seizure free (Engel I)	9	69.2	15	93.8
Continuing seizure (Engel II, III, IV)	4	30.8	1	6.3

HPE = histopathological examination; HS = hippocampal sclerosis; EEG = electroencephalogram; PET = positron emission tomography

**Table 11:** Seizure outcome for HS and tumour

HPE	Timeline	Percentage of seizure free group (Engel I)	*n*
HS	Immediate	92.3	12
	Three to six months	69.2	9
	One year	69.2	9

Tumour	Immediate	100.0	16
	Three to six months	93.8	15
	One year	93.8	15

HPE = histopathological examination; HS = hippocampal sclerosis

**Table 12:** HS vs tumour

Variable	HS	Tumour	*P*-value
Age at surgery (years)	12.9 (3.73)	11.5 (4.36)	0.365
Age on set of seizure (years)	6.1 (3.37)	7.0 (4.83)	0.547
Weight (kg)	53.0 (16.65)	42.3 (21.56)	0.156
Operation (hours)	4.8 (0.66)	5.0 (0.70)	0.398
Blood loss (%)	10.0 (4.50)	11.5 (6.25)	0.441
Number of medications pre-op	3.0 (1.00)	2.0 (1.00)	0.118
Number of medications post op	2.0 (1.00)	2.0 (1.00)	0.037
Post-operation length of stay (days)	3.0 (1.00)	3.0 (1.00)	0.462

* numerical data presented as Mean (SD) for normally distributed and Median (IQR) if skewed;

HS = hippocampal sclerosis

**Table 13:** Complications

Complications	*n* (%)
Left hemiparesis	1 (3.6)
Right hemiparesis	1 (3.6)
Right hemiparesis, left mydriasis and ptosis	1 (3.6)
Right hemiparesis and right nasal hemianopsis	1 (3.6)

Total	4 (14.4)

**Table 14:** Spearman’s correlation coefficient

Variable	*P*-value	Spearman’s correlation coefficient, *r*
Number of antiepilepsy before and after operation	0.021	0.427

**Table 15:** Wilcoxon Signed Rank test

Number of epileptic medications	*Z*	*P*-value
Post-operation – pre-operation	−2.724	0.006

**Table 16:** Reoperation characteristics

Age (year)	Preop MRI	Procedure	Pathology	Postop MRI	Reoperation	Seizure control
17	Left temporal neocortical lesion	ATL	Ganglioglioma	Incomplete resection	Completion of ATL	Engel I
16	Right temporal lesion	ATL	Low grade astrocytoma	Incomplete resection	Completion of ATL	Engel I

ATL = anterior temporal lobectomy
